# Evaluation of modified carbapenem inactivation method for suspected carbapenemase among *Enterobacteriaceae* clinical isolates

**DOI:** 10.18632/oncotarget.25603

**Published:** 2018-06-26

**Authors:** Beiwei Yu, Dong Dong, Mingxia Wang, Yan Guo, Dandan Yin, Fupin Hu

**Affiliations:** ^1^ Department of Laboratory Medicine, Jinhua People’s Hospital, Zhejiang Province, China; ^2^ Institute of Antibiotics, Huashan Hospital, Fudan University, Shanghai, China; ^3^ NHC Key Laboratory of Clinical Pharmacology of Antibiotics, Fudan University, Shangai, China; ^4^ The Clinical Microbiology Laboratory, Department of Nosocomial Infection Control, Children’s Hospital of Fudan University, Shanghai, China

**Keywords:** modified carbapenem inactivation method, carbapenemases, enterobacteriaceae, bla_KPC-2_, bla_NDM-1_

## Abstract

Modified carbapenem inactivation method (mCIM) testing was currently recommended by Clinical and Laboratory Standards Institute (CLSI) for detection of carbapenemase among *Enterobacteriaceae* clinical isolates. In this study, a panel of 145 clinical strains were collected for evaluating the mCIM for detection of carbapenemase. Antimicrobial susceptibility testing were performed by microbroth dilution and the results were interpreted according to CLSI guidelines. All strains were resistant to ertapenem with high MIC_50_ and MIC_90_ (64 mg/L –>128 mg/L). For *bla*_NDM-1_-positive or *bla*_OXA-232_-positive strains, the zone of inhibition of meropenem were all 6 mm despite the incubation time of 6 h, 18 h or 24 h. For 6 h, the zone of meropenem inhibition for most of carbapenemase-positive isolates were meet the positive criteria 6–15 mm. However, for carbapenemase-negative isolates, the zone of meropenem inhibition were 16–18 mm after 6 h incubation which should be considered indeterminate for standard incubating time such as 18 h or 24 h. After incubating for 18 h or 24 h, the zone of meropenem inhibition were 22–25 mm for carbapenemase-negative isolates and meet the negative criteria. Our study indicate mCIM is a simple and effective method to identify the carbapenemases producers among *Enterobacteriaceae* clinical isolates.

## INTRODUCTION

Production of carbapenemases are main mechanism underlying resistance to carbapenems which are first-line agents with proven efficacy for treatment of severe infections caused by multi-drugs resistant bacteria [1]. Carbapenemase-producing *Enterobacteriaceae* isolates are usually extensively drug resistant, and infections caused by these pathogens with significant morbidity and mortality present a serious clinical challenge, especially for pediatric patients [2]. Because genes mediated carbapenemase usually located in transferable plasmids and it can potentially spread rapidly, infections caused by carbapenemase-producing *Enterobacteriaceae* may prove difficult to control once they emerge [3]. For this purpose, rapid and reliable identification of carbapenemase producers in the clinical microbiology laboratory is urgently needed to affect infection treatment. Currently, although several commercial tests are available for detection of carbapenem resistance including phenotypic or genotypic tests, none is ideal for all possible carbapenemase genes [4]. In 2017, modified carbapenem inactivation method (mCIM) testing was recommended by Clinical and Laboratory Standard Institute (CLSI) for detection of carbapenemase among *Enterobacteriaceae* clinical isolates [5]. This method is simple, low-cost to assess phenotypic carbapenemase activity in *Enterobacteriaceae* and has been demonstrated a sensitivity >99% and specificity >99% for detection of KPC, NDM, VIM, IMP, SPM, SME and OXA-type carbapenemase [1]. In this study, we described a mCIM method with some modification for the identification of carbapenemase-producing *Enterobacteriaceae* clinical isolates.

## MATERIALS AND METHODS

### Strains

A panel of 145 clinical strains including 77 *bla*_KPC-2_-positive *K. pneumoniae*, 31 *bla*_NDM-1_-positive *K. pneumoniae*, 4 *bla*_NDM-1_-positive *E.coli*, 5 *bla*_OXA-232_-positive *K. pneumoniae*, and 28 carbapenem-susceptible *K. pneumoniae* were collected from four hospitals in China for evaluating the mCIM for suspected carbapenemase. All isolates were identified by vitek 2 compact system (BioMerieux, France), and the presence of carbapenemase genes was confirmed by specific PCR and sequence analysis. *E. coli* ATCC25922 was used as quality control strain in antimicrobial susceptibility testing. *E. coli* NCTC-13476 (*bla*_IMP_ positive) and *K. pneumoniae* NCTC-13440 (*bla*_VIM-1_ positive) were also used for positive control for mCIM.

### Antimicrobial susceptibility testing

Antimicrobial susceptibility testing were performed by microbroth dilution and minimum inhibitory concentrations (MICs) were interpreted according to CLSI guidelines [5].

### mCIM for suspected carbapenemase

mCIM for detection of carbapenemases among 145 *Enterobacteriaceae* clinical isolates were performed as described by CLSI [5]. The zone of inhibition of meropenem was recorded after incubating time for 6 h, 18 h, and 24 h, respectively.

## RESULTS

### Antimicrobial susceptibility testing

The results of antimicrobial agents susceptibility for all of 117 carbapenem-resistant *Enterobacteriaceae* isolates were detailed in Table 1. All strains were resistant to ertapenem with high MIC_50_ and MIC_90_ (64 mg/L and >128 mg/L), meropenem with high MIC_50_ and MIC_90_ (32 mg/L and 128 mg/L), however, 23.1%–49.6% of them were susceptible to ciprofloxacin, gentamicin and amikacin, respectively.

**Table 1:** Activities of various antimicrobial agents against 117 carbapenem-resistant *Enterobacteriaceae* clinical isolates

Antibiotic name	MIC Range (mg/L)	MIC_50_ (mg/L)	MIC_90_ (mg/L)	Resistant (%)	Susceptible (%)
Cefoperazone/Sulbactam	4 –>128	>128	>128	94.9	4.3
Piperacillin/Tazobactam	4 –>256	>256	>256	96.6	2.6
Cefazolin	64 –>128	>128	>128	100	0
Cefuroxime	32 –>128	>128	>128	100	0
Ceftazidime	1 –>128	>128	>128	97.4	2.6
Cefotaxime	1 –>128	>128	>128	97.4	1.7
Cefepime	4 –>128	128	>128	96.6	0
Ertapenem	1 –>128	64	>128	98.3	0
Imipenem	0.5 – 64	32	32	93.2	0.9
Meropenem	0.25 –>128	32	128	96.6	1.7
Amikacin	0.5 –>128	>128	>128	50.4	49.6
Gentamicin	0.25 –>128	128	>128	63.2	35
Ciprofloxacin	0.06 –>128	32	128	73.5	23.1

### mCIM for suspected carbapenemase production

**Figure 1:**
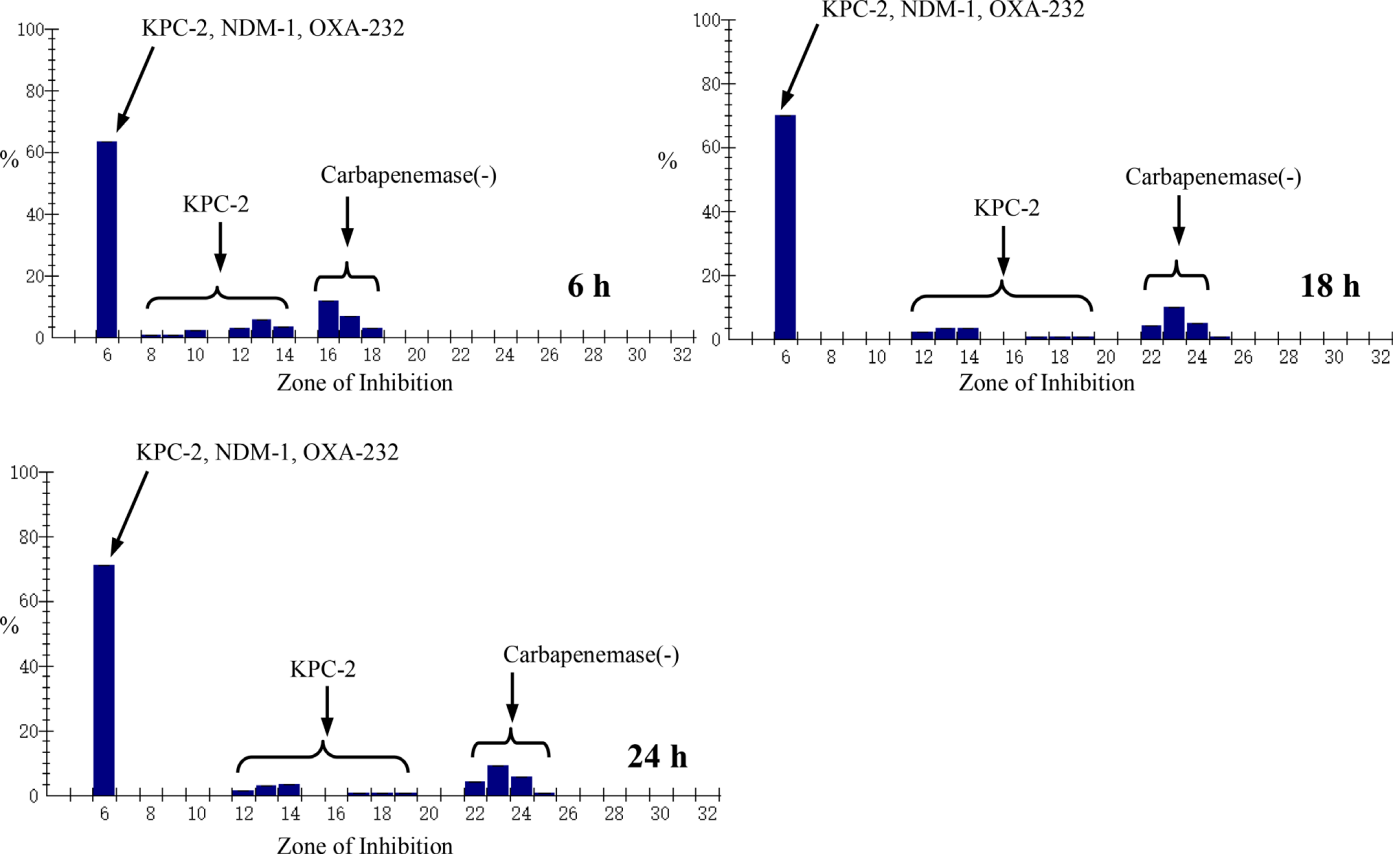
Zone of Inhibition distribution of meropenem for *Enterobacteriacae* clinical isolates at different incubating time

**Figure 2:**
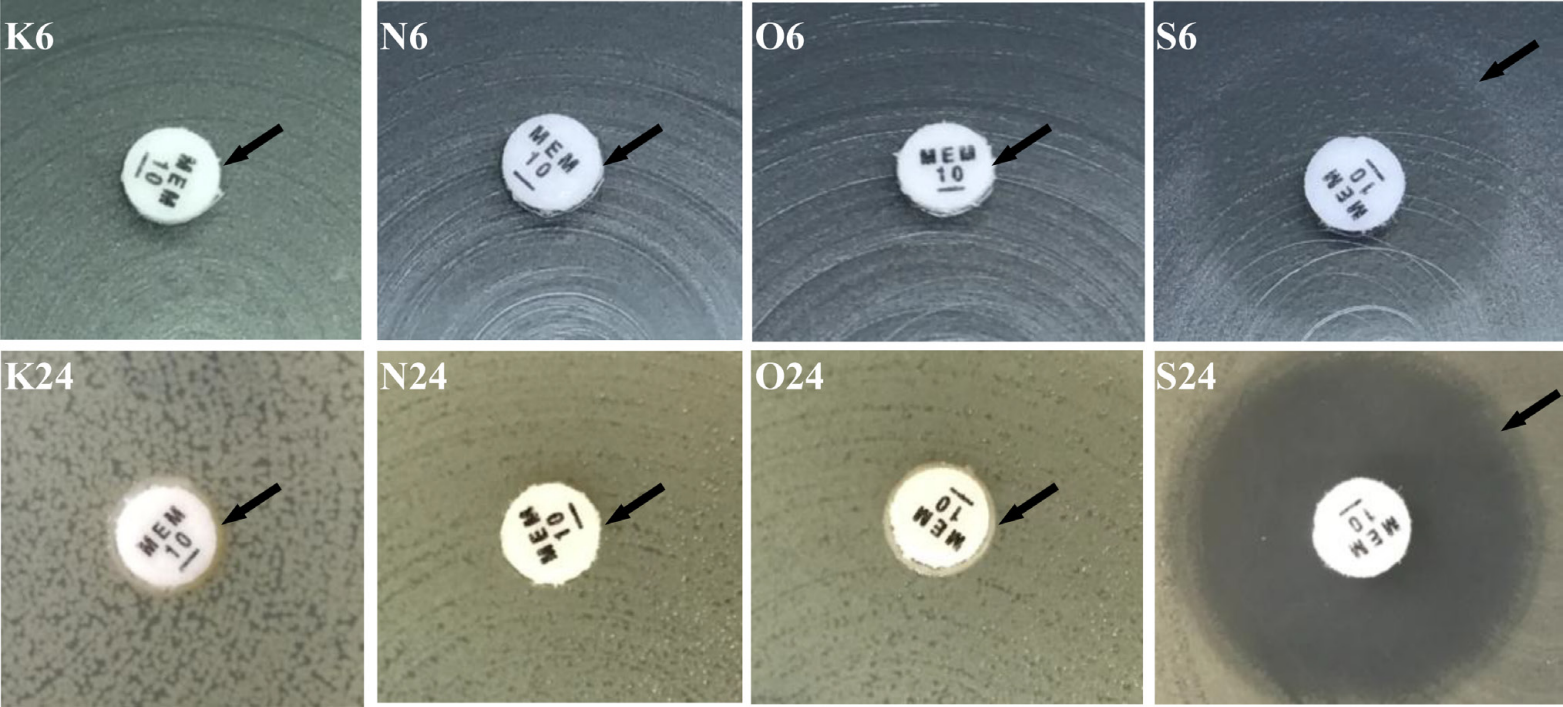
Modified carbapenem inactivation method for suspected carbapenemase production in enterobacteriaceae 1. K6, *bla*_kpc-2_ positive *K. pneumoniae* incubating 6 h (zone 6 mm); K24, *bla*_kpc-2_ positive *K. pneumoniae* incubating 24 h (zone 6 mm) (arrow indicated); 2. N6, *bla*_NDM-1_ positive *K. pneumoniae* incubating 6 h (zone 6 mm); N24, *bla*_NDM-1_ positive *K. pneumoniae* incubating 24 h (zone 6 mm) (arrow indicated); 3. O6, *bla*_OXA-232_ positive *K. pneumoniae* incubating 6 h (zone 6 mm); O24, *bla*_OXA-232_ positive *K. pneumoniae* incubating 24 h (zone 6 mm) (arrow indicated); 4. S6, meropenem-susceptible *K. pneumoniae* incubating 6 h (zone 18 mm); S24, meropenem-susceptible *K. pneumoniae* incubating 24 h (zone 22 mm) (arrow indicated).

Results obtained from mCIM indicated (Figures 1 and 2), for *bla*_NDM-1_-positive and *bla*_OXA-232_-positive strains, the zone of inhibition of meropenem were all 6 mm despite the incubation time of 6 h, 18 h or 24 h. For 18 h or 24 h, because of presence of colonies within a 16–18 mm meropenem zone for several *bla*_KPC-2_ producing *K. pneumoniae* isolates, mCIM can also differentiate successfully between carbapenemase-positive and carbapenemase-negative *Enterobacteriaceae* isolates. For 6 h, the zone of meropenem inhibition for all of carbapenemase-positive isolates were meet the positive criteria 6–15 mm. However, for carbapenemase-negative isolates, the zone of meropenem inhibition were 16–18 mm after 6 h incubation which should be considered indeterminate for standard incubating time such as 18 h or 24 h. After incubating for 18 h or 24 h, the zone of meropenem inhibition were 22–25 mm for carbapenemase-negative isolates and meet the negative criteria.

## DISCUSSION

In the past 10 years, the world-wide increase in carbapenem-resistant organisms has made it even more important to use these “last line” antibiotics which are the most broad-spectrum agents known and are often life-saving therapies for severe infections [6, 7]. According to CHINET surveillance, the resistance rate of *K. pneumoniae* isolates to carbapenem was increasing rapidly from 2005 to 2015 [1, 8]. Rapid and effective detection of carbapenemases is important for clinicians treating patients with these infections and for infection preventionists to limit the spread of carbapenem-resistant organisms [9]. mCIM recommended by CLSI in 2017 is a simple and inexpensive method to perform and is well established in many clinical microbiology laboratories based on its high sensitivity and specificity to detect carbapenemase-producing *Enterobacteriaceae* isolates compared with the current published or available phenotype method such as modified hodge testing and Craba-NP method [5]. Modified hodge testing is very simple to perform and no special reagents or media necessary. however, false-positive results can occur in isolates that produce ESBL or AmpC enzymes coupled with porin loss, and false-negative results are occasionally noted for some strains producing NDM carbapenemase. Carba_NP is a rapid method for detection of suspected carbapenemases among *Enterobacteriaceae*, but special reagents are needed, some of which necessitate inhouse preparation (and have a short shelf life). Given the rapid international spread of carbapenemase-producing isolates and the urgent need of treatment for the infection due to these isolates, a simple and effective mCIM for detection of KPC-2, NDM-1 and OXA-232-type carbapenemases which were the most common carbapenemases among *Enterobacteriacece* isolates in China [10, 11] is important.

In CLSI studies [5], one OXA-232-producing *K.pneumoniae* isolate was negative by mCIM at 4 out of validation sites, however, in this study, all of 5 OXA-232-producing *K.pneumoniae* isolates were positive by mCIM at different incubating time including 6 h, 18 h and 24 h. In this study, for 6 h, 18 h or 24 h, mCIM demonstrated a sensitivity 100% and specificity 100% for detection of KPC-2, NDM-1, OXA-232-type carbapenemases among *Enterobacteriaceae* isolates. For 6 h, the indeterminate results occurred for all of carbapenemase-negative *Enterobacteriaceae* isolates and the results indicated “testing inconclusive for the presence of carbapenemase”. So, for carbapenemase-negative *Enterobacteriaceae* isolates, it is necessary to extend the incubating time for confirming the production of carbapenemase.

Although carbapenemases involving in this study are only three type of carbapenemase. However, according to the previous studies, *bla*_KPC_ and *bla*_NDM-1_ are the main carbapenemase genes in our country [12]. Other genes including *bla*_OXA-48_, *bla*_IPM and_ *bla*_VIM_ are rare among *Enterobacterieceae* clinical isolates [13]. In some cases, especilly for pediatries infected by CRE, early and rapid detection of carbapenemase among *Enterobacterieceae* clinical isolates with 6 h incubation probably is vital the treatment with antimicrobial agents because the infection caused by carbapenem-resistant *Enterobacterieceae* clinical isolates are associated with significant morbidity and mortality [14, 15].
